# Supplementary material for: Adipose-Derived Mesenchymal Stem Cells do not Affect the Invasion and Migration Potential of Oral Squamous Carcinoma Cells
Source: Int J Mol Sci. 2020 Sep 4;21(18):6455. doi: 10.3390/ijms21186455 (PMC7555061; doi:10.3390/ijms21186455)
Supplement: Supplementary file 1 [file ijms-21-06455-s001.zip › Supplementary File 1.docx]

**Supplementary Table S1**. HSC-3 cells grown in conditioned media from AT-MSCs and BM-MSCs: quantitative PCR (raw data).

| **Conditioned media ^1^** | **Ct (mean)** | **Ct**  **(SD) ^2^** | ***RPLP0***  **Ct (mean) ^3^** | ***RPLP0* Ct (SD)** | **2^-ddCt^**  **^4^** | **2^-ddCt^** | | | |
| --- | --- | --- | --- | --- | --- | --- | --- | --- | --- |
|  |  |  |  |  |  | **mean** | | **SD** | |
| *ICAM1* | | | | | | | | | |
| Control | 23.822 | 0.030 | 17.443 | 0.123 | 1.000 | 1 | - | |  |
| A16-06 | 23.655 | 0.009 | 17.074 | 0.067 | 0.869 | 0.993 | 0.175 | |  |
| A18-01 | 23.174 | 0.065 | 16.954 | 0.077 | 1.117 |  |  |  |  |
| BMSC1608 | 24.439 | 0.084 | 17.436 | 0.140 | 0.649 | 0.700 | 0.072 | |  |
| BMSC1609 | 23.844 | 0.108 | 17.051 | 0.013 | 0.751 |  |  |  |  |
| *ITGA3* | | | | | | | | | |
| Control | 19.775 | 0.080 | 17.443 | 0.123 | 1.000 | 1 | - | |  |
| A16-06 | 19.521 | 0.078 | 17.074 | 0.067 | 0.923 | 0.979 | 0.079 | |  |
| A18-01 | 19.237 | 0.033 | 16.954 | 0.077 | 1.035 |  |  |  |  |
| BMSC1608 | 20.151 | 0.069 | 17.436 | 0.140 | 0.767 | 0.859 | 0.131 | |  |
| BMSC1609 | 19.455 | 0.086 | 17.051 | 0.013 | 0.952 |  |  |  |  |
| *MMP1* | | | | | | | | | |
| Control | 22.637 | 0.104 | 17.443 | 0.123 | 1.000 | 1 | - | |  |
| A16-06 | 22.094 | 0.119 | 17.074 | 0.067 | 1.128 | 1.395 | 0.377 | |  |
| A18-01 | 21.416 | 0.058 | 16.954 | 0.077 | 1.661 |  |  |  |  |
| BMSC1608 | 22.022 | 0.037 | 17.436 | 0.140 | 1.524 | 2.581 | 1.495 | |  |
| BMSC1609 | 20.382 | 0.151 | 17.051 | 0.013 | 3.638 |  |  |  |  |

^1^ A-prefixed samples were grown in AT-MSC derived conditioned media, while BMSC-prefixed samples were grown in BM-MSC derived conditioned media. Serum-free growth media was the control sample to whose Ct values the gene expression was normalized to.
^2^ SD = standard deviation
^3^ *RPLP0* is the housekeeping gene. Its Ct values are the same against each gene.
^4^ 2^-ddCt^ refers to the fold-change in gene expression. Calculations are not shown here but can be calculated from the provided data.

**Supplementary Table S2.** AT-MSCs and BM-MSCs grown in HSC-3 derived conditioned media: quantitative PCR (raw data).

| **Cell type and condition ^1^** | **Ct (mean)** | **Ct SD ^2^** | ***RPLP0***  **Ct (mean) ^3^** | ***RPLP0* Ct SD** | **2^-ddCt^**  **^4^** | **Mean**  **2^-ddCt^** | **SD**  **2^-ddCt^** |
| --- | --- | --- | --- | --- | --- | --- | --- |
| *ICAM1* | | | | | | | |
| A16-06 SF | 26.982 | 0.260 | 16.922 | 0.108 | 1.000 | 13.977 | 2.0x10^-14^ |
| A16-06 HSC-3 | 22.658 | 0.243 | 16.403 | 0.176 | **13.977** |  |  |
| A18-01 SF | 26.033 | 0.290 | 15.912 | 0.069 | 1.000 |  |  |
| A18-01 HSC-3 | 22.120 | 0.286 | 15.804 | 0.412 | **13.977** |  |  |
| BMSC1608 SF | 25.773 | 0.115 | 16.629 | 0.182 | 1.000 | 5.483 | 1.787 |
| BMSC1608 HSC-3 | 23.205 | 0.412 | 16.815 | 0.300 | **6.746** |  |  |
| BMSC1609 SF | 24.552 | 0.122 | 16.695 | 0.283 | 1.000 |  |  |
| BMSC1609 HSC-3 | 22.148 | 0.017 | 16.368 | 0.295 | **4.219** |  |  |
| *ITGA3* | | | | | | | |
| A16-06 SF | 26.946 | 0.468 | 16.922 | 0.108 | 1.000 | 1.041 | 0.123 |
| A16-06 HSC-3 | 29.020 | 3.329 | 16.403 | 0.176 | **0.954** |  |  |
| A18-01 SF | 27.248 | 0.025 | 15.912 | 0.069 | 1.000 |  |  |
| A18-01 HSC-3 | 26.967 | 0.044 | 15.804 | 0.412 | **1.127** |  |  |
| BMSC1608 SF | 22.909 | 0.037 | 16.629 | 0.182 | 1.000 | 0.803 | 0.281 |
| BMSC1608 HSC-3 | 23.093 | 0.187 | 16.815 | 0.300 | **1.001** |  |  |
| BMSC1609 SF | 22.009 | 0.146 | 16.695 | 0.283 | 1.000 |  |  |
| BMSC1609 HSC-3 | 22.410 | 0.258 | 16.368 | 0.295 | **0.604** |  |  |
| *MMP1* | | | | | | | |
| A16-06 SF | 30.400 | 0.130 | 16.922 | 0.108 | 1.000 | 32.843 | 34.958 |
| A16-06 HSC-3 | 26.859 | 0.287 | 16.403 | 0.176 | **8.124** |  |  |
| A18-01 SF | 30.123 | 0.316 | 15.912 | 0.069 | 1.000 |  |  |
| A18-01 HSC-3 | 24.167 | 0.185 | 15.804 | 0.412 | **57.562** |  |  |
| BMSC1608 SF | 32.305 | 0.340 | 16.629 | 0.182 | 1.000 | 31.267 | 35.506 |
| BMSC1608 HSC-3 | 26.674 | 0.098 | 16.815 | 0.300 | **56.373** |  |  |
| BMSC1609 SF | 27.358 | 0.215 | 16.695 | 0.283 | 1.000 |  |  |
| BMSC1609 HSC-3 | 24.408 | 0.090 | 16.368 | 0.295 | **6.161** |  |  |

^1^ SF refers to cells grown in serum-free media (control) and HSC-3 refers to cells grown in HSC-3 derived conditioned media.
^2^ SD = standard deviation
^3^ *RPLP0* is the housekeeping gene. Its Ct values are the same against each gene.
^4^ 2^-ddCt^ refers to the fold-change in gene expression. Calculations are not shown here.
