# Supplementary material for: Adipose-Derived Mesenchymal Stem Cells do not Affect the Invasion and Migration Potential of Oral Squamous Carcinoma Cells
Source: Int J Mol Sci. 2020 Sep 4;21(18):6455. doi: 10.3390/ijms21186455 (PMC7555061; doi:10.3390/ijms21186455)
Supplement: Supplementary file 1 [file ijms-21-06455-s001.zip › Supplementary File 1.pdf]

Supplementary Table S1. HSC-3 cells grown in conditioned media from AT-MSCs and BM-MSCs: quantitative PCR (raw data).

Conditioned media [1]	Ct (mean)	Ct (SD) [2]	*RPLP0* Ct (mean) [3]	*RPLP0* Ct (SD)	2^{-ddCt} [4]	2^{-ddCt} mean	2^{-ddCt} SD
ICAM1							
Control	23.822	0.030	17.443	0.123	1.000	1	-
A16-06	23.655	0.009	17.074	0.067	0.869	0.993	0.175
A18-01	23.174	0.065	16.954	0.077	1.117		
BMSC1608	24.439	0.084	17.436	0.140	0.649	0.700	0.072
BMSC1609	23.844	0.108	17.051	0.013	0.751		
ITGA3							
Control	19.775	0.080	17.443	0.123	1.000	1	-
A16-06	19.521	0.078	17.074	0.067	0.923	0.979	0.079
A18-01	19.237	0.033	16.954	0.077	1.035		
BMSC1608	20.151	0.069	17.436	0.140	0.767	0.859	0.131
BMSC1609	19.455	0.086	17.051	0.013	0.952		
MMP1							
Control	22.637	0.104	17.443	0.123	1.000	1	-
A16-06	22.094	0.119	17.074	0.067	1.128	1.395	0.377
A18-01	21.416	0.058	16.954	0.077	1.661		
BMSC1608	22.022	0.037	17.436	0.140	1.524	2.581	1.495
BMSC1609	20.382	0.151	17.051	0.013	3.638		

[1] A-prefixed samples were grown in AT-MSC derived conditioned media, while BMSC-prefixed samples were grown in BM-MSC derived conditioned media. Serum-free growth media was the control sample to whose Ct values the gene expression was normalized to.
[2] SD = standard deviation
[3] *RPLP0* is the housekeeping gene. Its Ct values are the same against each gene.
[4] 2^{-ddCt} refers to the fold-change in gene expression. Calculations are not shown here but can be calculated from the provided data.

Supplementary Table S2. AT-MSCs and BM-MSCs grown in HSC-3 derived conditioned media: quantitative PCR (raw data).

Cell type and condition [1]	Ct (mean)	Ct SD [2]	*RPLP0* Ct (mean) [3]	*RPLP0* Ct SD	2^{-ddCt} [4]	Mean 2^{-ddCt}	SD 2^{-ddCt}
ICAM1							
A16-06 SF	26.982	0.260	16.922	0.108	1.000	13.977	$2.0x10^{-14}$
A16-06 HSC-3	22.658	0.243	16.403	0.176	**13.977**		
A18-01 SF	26.033	0.290	15.912	0.069	1.000		
A18-01 HSC-3	22.120	0.286	15.804	0.412	**13.977**		
BMSC1608 SF	25.773	0.115	16.629	0.182	1.000	5.483	1.787
BMSC1608 HSC-3	23.205	0.412	16.815	0.300	**6.746**		
BMSC1609 SF	24.552	0.122	16.695	0.283	1.000		
BMSC1609 HSC-3	22.148	0.017	16.368	0.295	**4.219**		
ITGA3							
A16-06 SF	26.946	0.468	16.922	0.108	1.000	1.041	0.123
A16-06 HSC-3	29.020	3.329	16.403	0.176	**0.954**		
A18-01 SF	27.248	0.025	15.912	0.069	1.000		
A18-01 HSC-3	26.967	0.044	15.804	0.412	**1.127**		
BMSC1608 SF	22.909	0.037	16.629	0.182	1.000	0.803	0.281
BMSC1608 HSC-3	23.093	0.187	16.815	0.300	**1.001**		
BMSC1609 SF	22.009	0.146	16.695	0.283	1.000		
BMSC1609 HSC-3	22.410	0.258	16.368	0.295	**0.604**		
MMP1							
A16-06 SF	30.400	0.130	16.922	0.108	1.000	32.843	34.958
A16-06 HSC-3	26.859	0.287	16.403	0.176	**8.124**		
A18-01 SF	30.123	0.316	15.912	0.069	1.000		
A18-01 HSC-3	24.167	0.185	15.804	0.412	**57.562**		
BMSC1608 SF	32.305	0.340	16.629	0.182	1.000	31.267	35.506
BMSC1608 HSC-3	26.674	0.098	16.815	0.300	**56.373**		
BMSC1609 SF	27.358	0.215	16.695	0.283	1.000		
BMSC1609 HSC-3	24.408	0.090	16.368	0.295	**6.161**		

[1] SF refers to cells grown in serum-free media (control) and HSC-3 refers to cells grown in HSC-3 derived conditioned media.

[2] SD = standard deviation

[3] *RPLP0* is the housekeeping gene. Its Ct values are the same against each gene.

[4] 2^{-ddCt} refers to the fold-change in gene expression. Calculations are not shown here.
